# Response surface methodology for optimization of culture conditions for dye decolorization by a fungus, *Aspergillus niger* HM11 isolated from dye affected soil

**Published:** 2010-12

**Authors:** K Karthikeyan, K Nanthakumar, K Shanthi, P Lakshmanaperumalsamy

**Affiliations:** 1 Gujarat Institute of Desert Ecology, Mundra Road, Bhuj-Kachchh -370001, Gujarat, India.; 2 Department of Env. Sciences, Bharathiar University, Coimbatore-641 046, Tamil Nadu, India.; 3 Department of Env. Sciences, P.S.G.C.A.S, Coimbatore-641 014, Tamil Nadu, India.; 4 Karpagam University, Eachanari Post, Coimbatore-641 021, Tamil Nadu, India.

**Keywords:** Decolorization, Congo red, *Aspergillus niger* HM11, RSM, CCD

## Abstract

**Background and Objectives:**

Discharge of wastewater from textile dyeing industries has been a problem in terms of pollution and treatment of these waters is a great task. Keeping this in mind, the aim of our current research is to study the effect of various bioprocess variables on decolorization of an azo dye, Congo red, by a fungal isolate, *Aspergillus niger* HM11.

**Materials and Methods:**

Central composite design (CCD) and response surface methodology (RSM) have been applied to design experiments to evaluate the interactive effects of the operating variables: on the decolorization of Congo red. A total of 30 experiments were conducted in the present study and a regression coefficient between the variables was generated.

**Results:**

The RSM indicated that pH 6.0, 150 rpm agitation, incubation time of 36 hrs and a glucose concentration of 1.0% were optimal for maximum decolorization of Congo red and the response indicated excellent evaluation of experimental data.

**Conclusion:**

From this study, it is very obvious that the fungal isolate, *Aspergillus niger* HM11 can be used as a promising microbial strain for decolorization of textile dyeing effluent containing similar dyes.

## INTRODUCTION

Azo dyes are the most commonly used dyes in textile dyeing/finishing and also in food, paper and cosmetic industries. Approximately 10,000 different dyes and pigments are in industrial use, representing an annual consumption of around 7×10^5^ tonnes worldwide (1). According to the statistics, India, the former USSR, Eastern Europe, China, South Korea and Taiwan consume approximately one thousand tons (kt) of dye annually (2).

Color can be removed from effluents by chemical and physical methods including adsorption, coagulation-flocculation, ion-exchange, oxidation and electrochemical methods (3, 4). The above ways for clean-up are expensive, which limit their application (5). Over past decades, many microorganisms have been found to be capable of degrading dyes including bacteria (6, 7), filamentous fungi (8–10), yeasts (11, 12), actinomycetes (13) and algae (14). Anaerobic reduction and decolorization often generates aryl amines that can be transformed to highly reactive electrophiles and form covalent adducts with DNA, thereby posing a health risk (15, 16). Alternatively, dye decolorization using microbial enzymes has received great attention in recent years due to its efficient application (17, 18).

Fungi in general have proved to be a suitable organism for the treatment of textile effluent and dye removal. Minussi et al. (19) investigated the decolorization of Reactive Blue 19, Reactive Red195, Reactive Yellow 145 and Reactive Black 5 by four fungal strains: *Lentinus edodes* CCT 4519, *Trametes versicolor* CCT 4521, *Phanerochaete chrysosporium* ATCC 24725 and *Trametes villosa* CCT 5567. Combination of environmental conditions, culture technique and bioreactor design should be taken into account to produce high titres of enzyme (20). The fungal mycelia have an additive advantage over single cell organisms by solubilizing the insoluble substrates by producing extracellular enzymes and due to an increased cell-to-surface ratio, fungi have a greater physical and enzymatic contact with the environment. The extracellular nature of the fungal enzymes is also advantageous in tolerating high concentrations of the toxicants. However, it is reported that even low enzyme activity is sufficient to catalyze the decolourization of the dye solution (21).

Response surface methodology (RSM) is an efficient experimental strategy to determine optimal conditions for a multivariable system rather than optimization by the conventional method which involves changing one independent variable while keeping the other factors constant. These conventional methods are time-consuming and incapable of detecting the true optimum, especially the absence of interactions among factors (22) and in defining the effect of the independent variables, alone or in combination, on the processes (23, 24). Optimization of conditions for maximum removal of Congo red by statistical approach has been planned to determine the exact conditions for removal by *A. niger* HM11 which would be useful for industrial applications. Hence, the present study was aimed to examine the most influential variables for maximum decolorization of Congo red through Plackett -Burman design using*A. niger* HM11.

## MATERIAL AND METHODS

**Chemicals**. Dyes and other chemicals used in the experiments were purchased from Hi-Media, Mumbai and were of the highest purity.

**Microorganism**. The fungal strain was isolated from dye contaminated soil collected from a local textile industry situated in Coimbatore, Tamilnadu, India. Further, identification of the fungal isolate was carried out at Agharkhar Research Institute, Pune, India and was identified to be *A. niger* and designated as *A. niger* HM11.

**Culture conditions**. Sabouraud's dextrose agar (SDA) plates were prepared. Purified fungal isolates from slants were streaked onto SDA plates and incubated at 30°C for 5 days. After good growth, mycelial discs of 0.6 cm diameter were cut with a flame sterilized cork-borer and used as inoculum. For liquid cultures, Sabouraud's dextrose broth was used.

**Response surface methodology (RSM)**. RSM approach is used to generate the best conditions for a system comprising many variables to calculate the combined effect of selected variables (22–24). In the present study, RSM was employed to identify the interactions between the operational variables such as pH, temperature (°C), agitation (rpm), incubation time (hrs), dye concentration (mg/L), glucose concentration (%), peptone (%), magnesium sulphate (%), heavy metal chromium (mM), phenols (mM) and sodium (%) at various concentrations. We selected Plackett Burman design for the study on the interactions of different variables. Various concentrations of pH (3.0–11.0), agitation (0-300 rpm), incubation time (0–72 hrs) and gluc ose (0.1–1.0%) were chosen as the critical variables and dye decolorization experiments were carried out according to the arrangement presented in the Table 1.

**Table 1:** Plackett – Burman design for screening bioprocess variables affecting decolorization of Congo red.

Run	pH	Temp(°C)	Agitation (rpm)	Incubation time (hrs)	Dye concentration (mg/L)	Glucose (%)	Peptone (%)	Magnesium sulphate (%)	Chromium (mM)	Phenols (mM)	Sodium(%)
1	3	20	0	0	10	0.1	0.1	0.05	0.05	0.05	0.05
2	11	20	300	72	1000	0.1	0.1	0.05	0.5	0.05	0.5
3	11	60	0	0	10	1.0	0.1	0.5	0.5	0.05	0.5
4	11	20	300	72	10	1.0	0.5	0.5	0.05	0.05	0.05
5	3	20	0	72	10	1.0	0.5	0.05	0.5	0.5	0.5
6	11	20	0	0	1000	0.1	0.5	0.5	0.05	0.5	0.5
7	3	60	300	0	1000	1.0	0.5	0.05	0.05	0.05	0.5
8	11	60	0	72	1000	1.0	0.1	0.05	0.05	0.5	0.05
9	3	20	300	0	1000	1.0	0.1	0.5	0.5	0.5	0.05
10	11	60	300	0	10	0.1	0.5	0.05	0.5	0.5	0.05
11	3	60	0	72	1000	0.1	0.5	0.5	0.5	0.05	0.05
12	3	60	300	72	10	0.1	0.1	0.5	0.05	0.5	0.5

**Medium optimization for decolorization of Congo red by** ***A. niger*** **HM11**. The major conventional strategy used for optimal operating condition of a parameter is optimized by changing one parameter at a time and keeping the others at a constant level. This method often does not yield reliable results, is laborious, time consuming and impractical (22). In this regard, RSM is a useful model for studying the effect of several factors by varying them simultaneously and carrying out a limited number of experiments. This methodology consists of the Plackett – Burman design as the first optimization step, Central Composite design as a second step to optimize the factors that have significant effects, and response surface analysis.

**Screening of important nutrient components using Plackett – Burman design**. This study was done for screening medium components with respect to their main effects and not their interaction effects (25) on decolorization of Congo red by *A. niger* HM11. The medium components were screened for eleven variables at two levels; maximum and minimum using Plackett – Burman design. The design and levels of each variable is shown in Table 1. The medium was formulated as per the design and the flask culture experiments on dye decolorization was assayed as described earlier and the response was calculated as the rate of dye decolorization and expressed in percent.

The effect of each variable was calculated using the following equation; (25).

E is the effect of tested variable, M_+_ and M-are responses (dye decolorization) of trials at which the parameter was at its higher and lower levels respectively and N is the number of experiments carried out.The standard error (SE) of the variables was the square root of variance and the significance level (p – value) of each variable was calculated by using Student's t – test (E _xi_ is the effect of the tested variable).

**Optimization of concentrations of the selected varibles using RSM**. RSM approach was used to identify the optimum conditions for a multivariable system, and it can predict the combined effect of some variables. The screened medium components affecting dye decolorization were optimized using central composite design (CCD) (26, 27). According to this design, the total number of treatment combinations is 2^*k*^ +2*k+n*0 where ‘*k*’ is the number of independent variables and *n*_0_ is the number of experiment repetitions at the center point. For statistical calculation, the variables *Xi* have been coded as *xi* according to the following transformation (25):

$${\text{X}}_{\text{i}}={\text{X}}_{\text{i}}\_{\text{X}}_{\text{0}}/\delta \text{X}$$

where *x*_*i*_ is a dimensionless coded value of the variable *X*_*i*_, *X*_0_ is the value of the *X*_*i*_ at the center point, and δX is the step change. A 2^*k*^-factorial design with eight axial points and six replicates at the center point with a total number of 30 experiments was employed for optimizing the medium components (25).

The behavior of the system was explained by the following quadratic equation:

$$\text{Y}=\beta_{\text{0}}+\sum \beta_{\text{i}}{\text{X}}_{\text{i}}+\sum \beta_{\text{i}}{\text{X}}_{\text{i}}^{\text{2}}+\sum \beta_{\text{ij}}{\text{X}}_{\text{i}}{\text{X}}_{\text{i}}$$

where *Y* is the predicted response, *β*_0_ the intercept term, *β*_*i*_ is the linear effect, *β*_*ii*_ *is* the squared effect, and *β*_*ij*_ is the interaction effect (25). The statistical model was validated with respect to dye decolorization under the conditions predicted by the model in flask conditions. Samples were withdrawn at the desired intervals and dye decolorization was determined as described above.

## RESULTS

**Optimization of bioprocess variables using RSM and factorial design**. Plackett Burman design (25) was constructed to determine the nutritional requirements for color removal. Among the variables screened, the most influencing factors for decolorization with high significance level indicated by Pareto chart were in the order of agitation, incubation time, pH and glucose were identified and selected for further optimization.

**Fig. 1:**
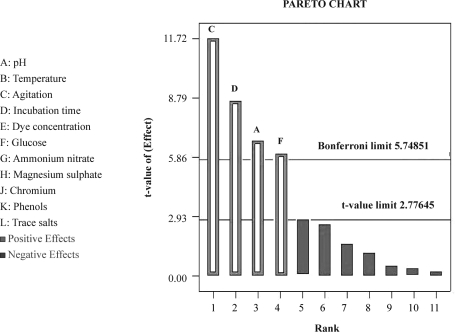
Pareto chart for Plackett Burman design for 11 factors on Congo red decolorization by *Aspergillus niger* HM11.

**Plackett -Burman design**. The influence of eleven factors (A–L) namely pH, temperature, agitation, incubation time, dye concentration, glucose, ammonium nitrate, magnesium sulphate, chromium, phenols and trace salts in the dye decolorization was investigated in 12 runs using Plackett – Burman design. Table 2 represents the Plackett–Burman design for 11 selected variables and the corresponding response for dye decolorization varied from 2.98 to 74.95%.The Pareto chart illustrates the order of significance (agitation, incubation time, pH and glucose) of the variables affecting dye decolorization (Fig. 1). In the present study, statistical analysis demonstrates that the model F value of 0.75 is significant. The values of p<0.05 indicate model terms are significant (Table 3). Regression analysis was performed on the results and first order polynomial equation was derived representing dye decolorization (Congo red) as a function of the independent variables.

**Table 2:** Plackett - Burman design for evaluating factors influencing Congo red degradation by *A. niger* HM11

Run	A	B	C	D	E	F	G	H	J	K	L	% decolorization
1	3	20	0	0	10	0.1	0.1	0.05	0.05	0.05	0.05	2.98
2	11	20	300	72	1000	0.1	0.1	0.05	0.5	0.05	0.5	71.21
3	11	60	0	0	10	1.0	0.1	0.5	0.5	0.05	0.5	7.75
4	11	20	300	72	10	1.0	0.5	0.5	0.05	0.05	0.05	74.95
5	3	20	0	72	10	1.0	0.5	0.05	0.5	0.5	0.5	6.04
6	11	20	0	0	1000	0.1	0.5	0.5	0.05	0.5	0.5	8.47
7	3	60	300	0	1000	1.0	0.5	0.05	0.05	0.05	0.5	65.42
8	11	60	0	72	1000	1.0	0.1	0.05	0.05	0.5	0.05	46.97
9	3	20	300	0	1000	1.0	0.1	0.5	0.5	0.5	0.05	30.66
10	11	60	300	0	10	0.1	0.5	0.05	0.5	0.5	0.05	54.45
11	3	60	0	72	1000	0.1	0.5	0.5	0.5	0.05	0.05	29.54
12	3	60	300	72	10	0.1	0.1	0.5	0.05	0.5	0.5	69.98

A: Ph B: Temperature (oC) C: Agitation (rpm) D: Incubation time (hrs) E: Dye CONCENTRATION (mg/L) F: Glucose (%) G: Peptone (%) H: Magnesium sulphate (%) J: Chromium (mM) K: Phenol (mM) L: Sodium salts (%)

**Table 3:** Analysis of variance for Congo red decolorization.

Source	Sum of square	Degree of freedom	Mean square	F–Value	*p-*Value	
Model	8571.060	7	1224.440	0.758	0.003	Significant
A-pH	291.856	1	291.856	6.855	0.059	
C-Agitation	5848.550	1	5848.550	137.365	0.000	
D-Incubation time	1385.890	1	1385.890	32.550	0.005	
F-Glucose	7.239	1	7.239	0.170	0.701	
Residual	170.307	4	42.577			
Cor Total	8741.360	11				

% Decolorization=39.04+4.93 A+22.08 C+10.75 D+3.01 F

The magnitude of the effects indicates the level of significance of the variable on decolorization of Congo red. Consequently, statistically significant variables with positive effect were further investigated to find the optimal range of these variables.**Central Composite Design (CCD)**. The result of 30 run CCD in four variables; pH, agitation, incubation time and glucose, chosen for optimization of dye decolorization process by *A. niger* HM11, are shown in Table 4. The table shows percent decolorization corresponding to combined effect of four components in their specified ranges.Decolorization varied markedly with the conditions tested, in the range of 0–99.8%. Lowest decolorization was observed when agitation was high with low pH value (run 4, 14 and 20). Decolorization value of 99.8% was observed at pH 6, agitation of 150 rpm, incubation time of 36 h and glucose of 1.0 g/L (run 29). The experimental results suggest that these variables strongly affect the decolorization process.

**Table 4:** Experimental plan for optimization of Congo red decolorization using central composite design (CCD).

					Decolorization (%)	
					
Run	pH	Agitation (rpm)	Incubation time (hrs)	Glucose (%)	Experimental	Predicted
1	0	0	0	0	82.60	95.62
2	−1	−1	−1	−1	45.80	51.09
3	0	0	0	−2	90.90	84.31
4	−1	1	1	−1	0.00	0.00
5	0	0	0	0	97.60	95.62
6	−1	1	−1	−1	20.80	32.72
7	0	2	0	0	38.90	29.18
8	0	0	0	0	98.90	95.62
9	0	0	0	0	97.60	86.16
10	1	−1	1	−1	32.50	46.69
11	0	0	−2	0	97.60	95.62
12	0	0	0	0	97.20	95.62
13	−1	1	−1	1	23.80	31.56
14	−2	0	0	0	0.00	0.00
15	−1	−1	1	−1	11.20	21.78
16	−1	−1	1	1	13.50	19.38
17	1	1	1	1	12.60	19.26
18	1	1	−1	−1	55.80	51.88
19	1	1	1	−1	55.80	39.87
20	2	0	0	0	0.00	0.00
21	−1	−1	−1	1	12.80	12.43
22	1	−1	−1	−1	32.50	37.25
23	−1	1	1	1	15.80	27.05
24	1	−1	1	1	15.80	6.58
25	0	0	0	2	96.40	85.04
26	0	0	2	0	97.60	61.09
27	0	−2	0	0	95.10	86.88
28	1	−1	−1	1	12.90	22.89
29	0	0	0	0	12.90	95.62
30	1	1	−1	1	2.60	11.02

The results obtained were subjected to analysis of variance with the regression model given as:

Y = 95.62 + 2.09 A−4.43 B−3.77 C -4.82 D + 3.25 AB + 2.19 AC−6.42 A D−0.36 BC + 1.88 B D + 4.06 C D−31.15 A^2^ -14.40 B^2^−6.75 C^2^−7.74 D^2^

where, Y is the response value (% decolorization) and A, B, C and D are the coded levels of pH, agitation, incubation time and glucose concentration, respectively. The adequacy of the model was checked using analysis of variance and the results are presented in Table 5.

**Table 5:** ANOVA for the experimental results of the central composite design (quadratic model)

Source	Sum of square	Degree of freedom	Mean square	F–Value	*p-*Value	
Model	32221.200	14	2301.511	2.728	0.032	Significant
A-pH	105.002	1	105.002	0.124	0.729	
B-Agitation	469.935	1	469.935	0.557	0.467	
C-Incubation time	340.507	1	340.507	0.404	0.535	
D-Glucose	556.807	1	556.807	0.660	0.429	
AB	169.000	1	169.000	0.200	0.661	
AC	76.563	1	76.563	0.091	0.767	
AD	660.490	1	660.490	0.783	0.390	
BC	2.103	1	2.103	0.002	0.961	
BD	56.250	1	56.250	0.067	0.800	
CD	264.063	1	264.063	0.313	0.584	
A2	26611.000	1	26611.000	31.538	< 0.0001	
B2	5685.940	1	5685.943	6.739	0.020	
C2	1248.940	1	1248.943	1.480	0.243	
D2	1641.230	1	1641.234	1.945	0.183	
Residual	12656.800	15	843.788	–	–	
Lack of Fit	12448.700	10	1244.874	29.912	0.001	Significant
Pure Error	208.088	5	41.618	–	–	
Cor Total	44878.000	29	–	–	–	

CV-6.03; R^2^-0.7180

**Fig. 2:**
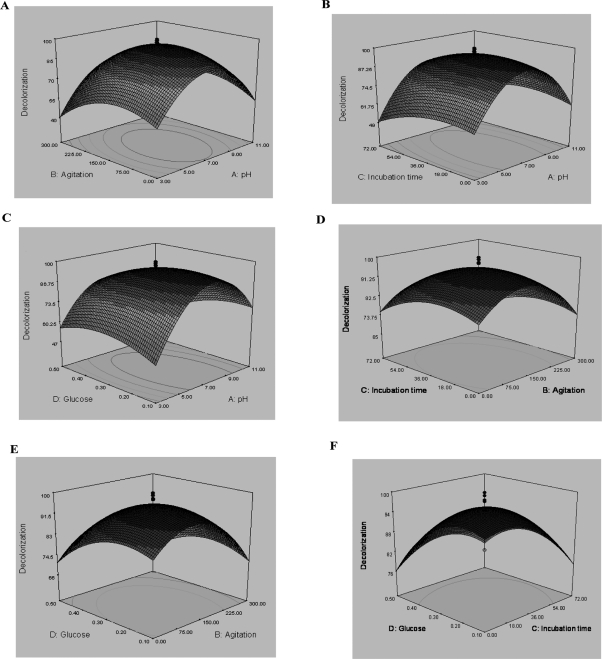
Three dimensional response surface plot for the effect of (A) pH, agitation; (B) pH, incubation time; (C) pH, glucose; (D) agitation, incubation time; (E) agitation, glucose; (F) incubation time, glucose on decolorization of Congo red by Aspergillus niger HM11

Interaction effects and optimal levels of the variables were determined by plotting the response surface contour plots (Fig. 2) which showed the behavior of response (% decolorization) with respect to simultaneous change in two variables.

**Fig. 3:**
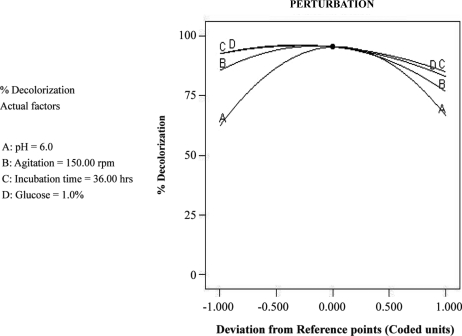
Perturbation graph showing the optimum values of the tested variables.

**Validation of the model**. The application of an experimental design for optimization of the decoloriza- tion ability of fungal strain *A. niger* HM 11 on Congo red was performed. The maximum experimental response for dye decolorization was 99.80% whereas the predicted value was 95.62% indicating a strong concurrence between them. The optimum values of the tested variables are pH (6.0), agitation (150 rpm), incubation time (36 hrs) and glucose (1.0%) as shown in perturbation graph (Fig. 3). In this case, the value of the determination coefficient indicates the reliability of the model.

## DISCUSSION

Microorganisms are capable of utilizing a variety of complex chemicals including dyes as their sole source of carbon but only few researchers have been successful in isolating such culture (28). Among the variables screened, the most effective factors with high significance level indicated by Pareto chart were agitation, incubation time, pH and glucose, in that order. In line with our studies, Mohana *et al*. (29) observed glucose was the best carbon source supporting maximum decolorization of anaerobically treated distillery effluent. Similar reports were given by Thongchai and Worrawit (30) reporting decrease in decolorization pattern of Fast red (FR) might be due to the reduction reaction involving the nitrogen in the medium and the nitrogen in the dyes and they have stated that increase in glucose, KH_2_PO_4_, MgSO_4._7H_2_O, and NaCl levels had a positive effect on FR decolorization,whereas, contrarily, increase in (NH_4_)_2_SO_4_ and K_2_HPO_4_ concentrations led to a negative effect on decolorization of FR.

Data of Table 4 shows percent decolorization corresponding to combined effect of four components in their specified ranges. The analysis of variance of the quadratic regression model suggested that the model is very significant as was evident from the Fisher's F – test. The R^2^ value (multiple correlation coefficient) closer to 1 denotes better correlation between the experimental and predicted responses. In the present case, a low CV (6.03) denotes the experiments performed are reliable. The *p* value denotes the significance of coefficients and is also important in understanding the pattern of mutual interactions between the variables. Similar R^2^ values were reported which confirmed that coefficient of determination (R^2^) equation was highly reliable and can be concluded that such studies are therefore very useful to predict variables for effective dye removal (31).

The 3D response surface plots described by the regression model were drawn to illustrate the effects of the independent variables and combined effects of each independent variable upon the response variable. In line with the present findings, similar reports on such decolorization pattern with similar bioprocess variables was observed during decolorization of 100 mg/L of Reactive Blue-25 by *A. ochraceus* NCIM-1146 at pH 5.0 (32), agitation speed of 100–150 rpm influenced better decolorization of Astrazon red FBL by *Funalia trogii* (33). Tatarko and Bumpus (34) showed 500mg/L of Congo red was 70% decolorized in 2 days by *Phanerochaete chrysosporium*. Similar to the present study, 3.0 g/L of glucose was used for decolorization of textile wastewater employing *A. niger* to obtain maximum decolorization (35).

The overall results show that this fungus has great possibilities to decolorize the textile dyes present in the effluents of textile industries and further reactor scale studies are required for actual industrial applications.
